# How to reward animals based on their subjective percepts: A Bayesian approach to online estimation of perceptual biases

**DOI:** 10.1101/2024.07.25.605047

**Published:** 2024-07-25

**Authors:** Yelin Dong, Gabor Lengyel, Sabyasachi Shivkumar, Akiyuki Anzai, Grace F. DiRisio, Ralf M. Haefner, Gregory C. DeAngelis

**Affiliations:** 1 Department of Brain and Cognitive Sciences, University of Rochester, Rochester, NY, USA; 2 Zuckerman Institute, Columbia University, New York, NY, USA

## Abstract

Elucidating the neural basis of perceptual biases, such as those produced by visual illusions, can provide powerful insights into the neural mechanisms of perceptual inference. However, studying the subjective percepts of animals poses a fundamental challenge: unlike human participants, animals cannot be verbally instructed to report what they see, hear, or feel. Instead, they must be trained to perform a task for reward, and researchers must infer from their responses what the animal perceived. However, animals’ responses are shaped by reward feedback, thus raising the major concern that the reward regimen may alter the animal’s decision strategy or even intrinsic perceptual biases. We developed a method that estimates perceptual bias during task performance and then computes the reward for each trial based on the evolving estimate of the animal’s perceptual bias. Our approach makes use of multiple stimulus contexts to dissociate perceptual biases from decision-related biases. Starting with an informative prior, our Bayesian method updates a posterior over the perceptual bias after each trial. The prior can be specified based on data from past sessions, thus reducing the variability of the online estimates and allowing it to converge to a stable estimate over a small number of trials. After validating our method on synthetic data, we apply it to estimate perceptual biases of monkeys in a motion direction discrimination task in which varying background optic flow induces robust perceptual biases. This method overcomes an important challenge to understanding the neural basis of subjective percepts.

## 2 Introduction

**Figure 1:**
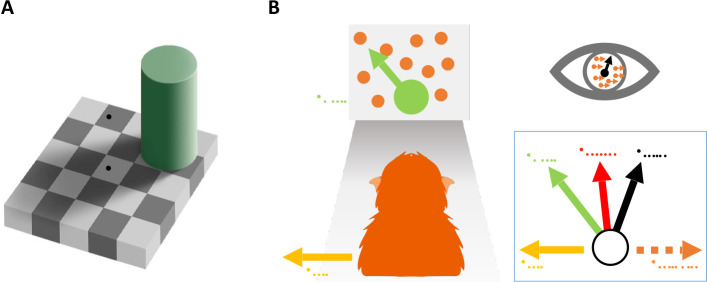
Two examples of how contextual information can bias visual perception. **A:** Luminance illusion created by shadows (Adelson, 1995). Square **B** looks brighter than square **A** but has the same luminance, i.e., they have identical grayscale values in the picture. **B:** Perception of object motion is biased by self-motion (Fajen & Matthis, 2013; Peltier et al., 2020; Rushton & Warren, 2005; Warren & Rushton, 2008, 2009a, 2009b). If the animal partially subtracts the optic flow vector (orange dashed arrow, *v*_*opticflow*_) generated by self-motion (yellow arrow, *v*_*self*_) from the image motion on the retina (black arrow, *v*_*retina*_), they may have a biased perception of object motion (red arrow, *v*_*percept*_) that lies between retinal and world coordinates (green arrow, *v*_*world*_).

In the natural environment, our subjective percepts often deviate from the sensory information entering our nervous system. Contextual information shapes perception of even low-level features, such as luminance (Adelson, 1993, 2000; Paradiso & Nakayama, 1991), and continues to modulate perception along the processing hierarchy (Bar, 2004; Oliva & Torralba, 2007). Some of the most compelling examples of biased perception are visual illusions. For example, in the classic Checkerboard illusion (Adelson, 1995), we perceive the ”white” square shadowed by the cylinder (square B in Fig. 1A) to be brighter than the black square outside of the shadow (square A in Fig. 1A), although the two squares have identical luminance. A systematic bias in behavior that originates from perceptual processes is called a *perceptual bias* (Raslear, 1985). These perceptual biases are typically quantified using decision-making tasks that measure the preference of an observer to choose one option over another (e.g., square B appears brighter/darker than square A).

Studying perceptual biases has been of great interest because it can provide insight into the underlying perceptual and cognitive processes. For example (Fig. 1B), by measuring perceptual biases in motion perception, prior work has revealed that both human and animal observers judge object motion by subtracting (a portion of) the optic flow due to self-motion from the retinal flow field (Fajen & Matthis, 2013; Peltier et al., 2020; Rushton & Warren, 2005; Warren & Rushton, 2008, 2009a, 2009b).

To study the neural basis of perceptual biases, animal models are particularly valuable as they provide rich electrophysiological data through invasive techniques (Fang et al., 2019; Peltier et al., 2024). However, measuring perceptual biases in animals poses a fundamental challenge (Beran & Parrish, 2022). Whereas humans can be verbally instructed to report what they see, hear, or feel without receiving feedback, animals must be trained to perform a task in exchange for some form of reward. A central challenge is that animals’ behavioral reports are shaped by the reward feedback they receive (Feng et al., 2009; J. Gao et al., 2011; Rorie et al., 2010). As a result, if stimulus context biases perception away from the rewarded response defined in the task, animals may learn to compensate for their biased subjective percepts to receive more rewards (Peltier et al., 2020). This concern is especially acute for neuroscientific studies since animals often need to perform a task for many thousands of trials and rewards must be provided continuously to keep the animals motivated. Our goal here is to estimate perceptual biases online and reward animals such that they won’t learn to compensate for their intrinsic perceptual biases based on reward feedback.

In previous studies, researchers followed a few different strategies for rewarding animals when the subjective percept was expected to deviate from the true stimulus value (Agrillo et al., 2015; Agrillo et al., 2014; Barbet & Fagot, 2002, 2007; Bayne & Davis, 1983; Beran & Parrish, 2022; Clara et al., 2006; Fujita, 1997; Huang et al., 2002; Parrish et al., 2015; Tudusciuc & Nieder, 2010), which we shall refer to as a ”bias context”. In some studies, animals were always rewarded on bias context trials to prevent the animals from compensating for their bias to receive more rewards (e.g., Bayne and Davis, 1983; Fujita, 1997). In other studies, researchers never rewarded animals on bias context trials (e.g., Agrillo et al., 2015; Agrillo et al., 2014; Clara et al., 2006). Other strategies involve rewarding the animal randomly (e.g., Barbet and Fagot, 2002, 2007) or with some fixed rate (e.g., Huang et al., 2002; Peltier et al., 2020) on bias context trials, and some studies just reward animals for veridical performance, assuming no perceptual bias (e.g., Parrish et al., 2015; Tudusciuc and Nieder, 2010). These variations in reward strategy may lead to large individual variability in the sign, pattern, and extent of measured perceptual biases (Beran & Parrish, 2022).

Most of these previous animal studies of illusions focus on behavior, and the animals are typically only required to complete tens or hundreds of trials (e.g., Agrillo et al., 2015; Agrillo et al., 2014; Bayne and Davis, 1983; Fujita, 1997; Huang et al., 2002). In such cases, animals may not learn to compensate for their perceptual biases due to limited exposure to illusion/bias trials. In many neuroscience experiments that involve electrophysiology, however, animals need to perform tasks over long periods of time (several months), often involving tens of thousands of trials. In this scenario, all of the above-mentioned approaches to rewarding animals in bias contexts become problematic, as animals have ample opportunity to learn to compensate for their perceptual biases to maximize reward. Indeed, a recent study reported that the perceptual biases of two macaque monkeys decreased over weeks and months in a motion discrimination task that invoked flow parsing (Peltier et al., 2020). Thus, there is a critical need for an approach to estimate perceptual biases online and to reward animals around their intrinsic biases, thereby removing the drive to compensate in order to maximize reward.

We developed a method that first infers the animal’s biased percept in each trial and then provides a reward based on what the animal most likely perceived. This approach requires the researcher to infer the perceptual biases of the animal online after each trial, which is challenging for the following reasons. First, in perceptual decision-making tasks, it is difficult to dissociate a perceptual bias from other decision- and response-related biases because the overall bias in the animal’s responses reflects the net result of all perceptual, cognitive, and response-related processes. To disentangle these processes, previous studies attempted to dissociate the origins of response biases (Cicchini et al., 2021; Cicchini et al., 2017; Drugowitsch et al., 2016; Fritsche et al., 2017; Linares et al., 2019; Zamboni et al., 2016). In the case of the widely-used two alternative forced-choice (2AFC) task, it is impossible to determine whether a response bias is due to perceptual bias, or a decision bias (Drugowitsch et al., 2016) without using multiple different tasks (Jazayeri & Movshon, 2007; Sánchez-Fuenzalida et al., 2023; Zamboni et al., 2016), or task conditions (Cicchini et al., 2021; Cicchini et al., 2017; Fritsche et al., 2017; Linares et al., 2019) that are interleaved trial by trial.

Second, even with an approach to separate perceptual biases from other decision-related biases, accurately estimating the perceptual bias from a small number of trials is difficult. The statistically optimal method for estimating the value (and uncertainty) of a latent variable (perceptual bias) from noisy measurements (responses of the animal) is Bayesian inference. This method combines prior beliefs about the values and the likelihood of the data given the values of the latent variable (Dempster, 1968; O’Reilly et al., 2012). Thus, with sufficiently informative prior beliefs, it should be possible to obtain useful estimates of the perceptual bias from a small number of trial outcomes.

In this study, we developed a Bayesian method that makes use of multiple stimulus conditions to perform online estimation of perceptual biases separately from other decision-related biases. We demonstrate the validity of our approach using ground-truth simulations and also apply it to the behavior of macaque monkeys performing a motion discrimination task. Our method allows us to estimate monkeys’ perceptual biases after each trial and allocate rewards accordingly. In contrast to a previous study that used a random reward strategy in bias context trials (Peltier et al., 2020), we show that an animal’s perceptual biases remained stable across more than 50 training sessions, thus demonstrating the efficacy of our approach.

## 3 Results

### 3.1 Rewarding Animal Behavior Relative to Perceptual Biases

**Figure 2:**
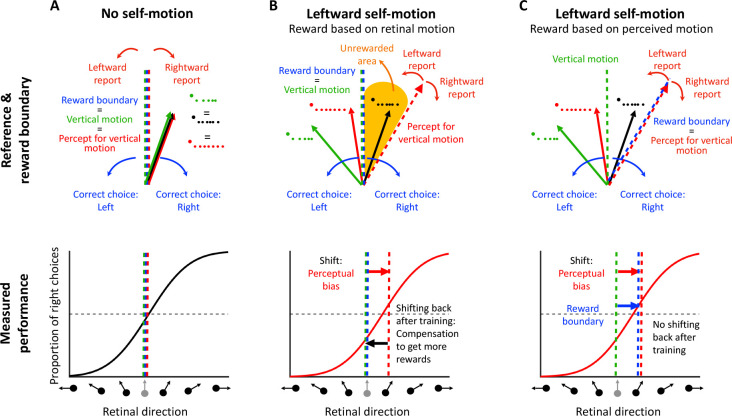
Reward strategies for a motion discrimination task with simulated self-motion. **A, Top:** No self-motion: the perceived direction (*v*_*percept*_, red arrow) matches both the retinal (*v*_*retina*_, black arrow) and the world directions (*v*_*world*_, green arrow). The vertical task reference (vertical motion, green dashed line) and perceived vertical motion (red dashed line) are aligned. Therefore, rewarding the animal veridically (reward boundary, blue dashed line) will not induce a perceptual bias. **A, bottom:** The corresponding psychometric curve shows the proportion of ”right” choices (y-axis) as a function of the retinal motion direction (x-axis), which equals the object motion in the world. The psychometric curve shows no horizontal shift (perceptual bias, red dashed line) because the retinal, world, and perceived motion directions are the same. **B, Top:** Leftward self-motion associated with rightward optic flow: the perceived direction (*v*_*percept*_, red arrow) is likely to be shifted leftward relative to motion on the retina (*v*_*retina*_, black arrow), and rightward relative to motion in the world (*v*_*world*_, green arrow). If the animal is rewarded for discriminating direction relative to a vertical reference (blue dashed line), there will be a range of directions that the animal perceives as leftward but will not be rewarded (yellow area). **B, Bottom:** The psychometric curve in the leftward self-motion condition is expected to be shifted to the right, reflecting the perceptual bias of the animal (red arrow). However, with extensive training on the task, the animal is likely to adopt a compensatory strategy that shifts the psychometric curve back to the left, such that the overall response bias no longer reflects the underlying perceptual bias. **C, Top**: If the reward boundary (blue dashed line) is rotated to match the animal’s percept for vertical motion (red dashed line), then the unrewarded area is eliminated. **C, Bottom**: Using this reward strategy, the intrinsic perceptual bias of the animal can be measured from the psychometric curve even after extensive training on the task.

First, we illustrate the problem of rewarding animals based on what they perceive, using the example of object motion perception in the context of self-motion. When there is no self-motion, i.e., the animal is stationary in the world (see Fig. 2A), there is no contextual information that biases perception. Thus, the perceived object motion (*v*_*percept*_) matches (on average) the actual object motion in the world (*v*_*world*_) and what is displayed on the screen (*v*_*retina*_). In this case, the vertical task reference (green dashed lines in Fig. 2) and the motion direction that the animal perceives as vertical (red dashed lines in Fig. 2) are aligned. Under these conditions, rewarding the animal is straightforward: you simply reward them in accordance with retinal or world motion (which are the same in this case). This is a common scenario in many 2AFC tasks that are performed by animals (e.g., Britten et al., 1992; Purushothaman and Bradley, 2004), in which no perceptual bias is expected such that the researcher-imposed reward boundary (blue dashed lines in Fig. 2) and the task reference (green dashed lines) are the same. In this case, the proportions of leftward and rightward responses are expected to be equal at the task reference direction, resulting in a psychometric curve that is centered at the vertical reference (Fig. 2A, bottom).

However, it is well established that background optic flow consistent with self-motion can bias the perception of object motion (Fajen & Matthis, 2013; Peltier et al., 2020; Rushton & Warren, 2005; Warren & Rushton, 2008, 2009a, 2009b). Consider an example (Fig. 2B, top) in which the animal is exposed to rightward background optic flow that simulates leftward self-translation, while the target object is moving up and to the left in the world (green vector, *v*_*world*_). This combination produces image motion of the object that is up and to the right (black vector, *v*_*retina*_). Previous studies have shown that the object motion perceived by humans (red vector, *v*_*percept*_) typically lies between retinal velocity, *v*_*retina*_, and object velocity in the world, *v*_world_ (Fajen & Matthis, 2013; Peltier et al., 2020; Rushton & Warren, 2005; Warren & Rushton, 2008, 2009a, 2009b). If the animal was trained to discriminate motion relative to a vertical reference in screen coordinates, then flow parsing would introduce a leftward perceptual bias in behavioral reports (in retinal coordinates). This perceptual bias can be observed as a horizontal shift in the psychometric curve (red arrow in Fig. 2B, bottom).

If the animal is rewarded for reporting object motion relative to the vertical reference direction (blue and green dashed vertical lines in Fig. 2B, top), there will be a subset of conditions in which the animal perceives the object motion as leftward but is rewarded for choosing rightward (orange area in Fig. 2B, top). To maximize rewards, the animal may learn to compensate for their perceptual bias and report a motion direction opposite to what they perceive. After an extensive training period, the shift in the psychometric curve may gradually diminish as a result of this compensation process (black arrow in Fig. 2B, C, bottom). In extreme cases, the curve may even return to the center, thus eliminating the response bias of the animal. In this case, the researcher may incorrectly conclude that there was no perceptual bias induced by optic flow.

A solution to this problem entails rewarding animals for reporting their subjective perception rather than the veridical stimulus value in retinal coordinates. In our task example, if we shift the reward boundary to align with the animal’s subjective percept of vertical motion (red dashed line in Fig. 2B, C, top), the unrewarded area will disappear and the animal will be rewarded for reporting direction relative to its subjective vertical. With this reward boundary, rewards will not influence the animal’s intrinsic perceptual bias, because the reward rate is maximized. Even after extensive training, the animal’s perceptual bias is expected to persist, and thus researchers should be able to measure it as a horizontal shift in the psychometric curve (in retinal coordinates). Thus, if an animal’s perceptual bias can be reliably estimated during the task, then rewarding the animal relative to their intrinsic perceptual bias should maintain stable performance over time.

### 3.2 Disentangling Perceptual and Decision Biases Using a Bayesian Approach

**Figure 3:**
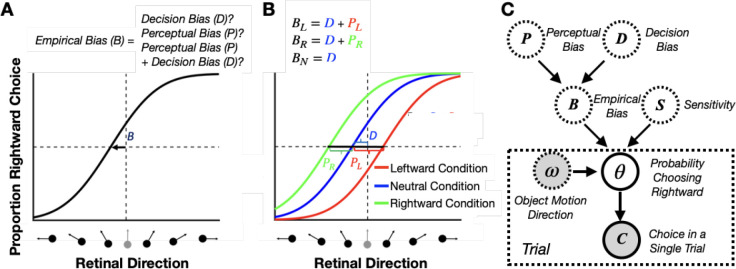
Disentangling perceptual and decision biases. **A:** Decision-related biases also shift the psychometric function horizontally in 2AFC tasks. From a single psychometric curve, it is impossible to know whether the empirically measured shift, B, was a decision bias, a perceptual bias, or a combination of the two. **B:** Separating perceptual and decision biases with multiple stimulus conditions in the case of judging object motion during self-motion. Equations show how to compute the perceptual ($P_{R}$ and $P_{L}$) and decision (D) biases from the empirically measured biases ($B_{L},\;B_{R}$ and $B_{N}$) in three stimulus conditions with leftward, rightward, and no self-motion, respectively. We assume that the decision-related bias (D) is constant across the three interleaved conditions. **C:** The generative model of our Bayesian approach for estimating perceptual and decision biases. We assume that the subject has a probability θ of choosing rightward motion relative to the reference for each motion direction. The percentage of ”right” choices across all trials in one session follows a binomial distribution, characterized by the probability θ of choosing rightward motion and number of trials n. The psychometric curve reflects the relationship between θ and the object direction ω, often described using a cumulative Gaussian distribution. The sensitivity S and the empirical bias B influence the slope and shift of the psychometric curve, respectively. Empirical bias for each condition B is determined by the perceptual bias P and decision bias D variables.

Rewarding animals for reporting their subjective percepts is only possible if the animal’s perceptual bias can be reliably estimated. In general, perceptual biases cannot simply be measured as the shift of the psychometric curve. Since the psychometric curve reflects both perceptual and decision-related processes, attributing the cause of the shift only to biased perception is impossible in most 2-AFC tasks (Drugowitsch et al., 2016; Garcia-Pérez & Alcalá-Quintana, 2013; Jogan & Stocker, 2014; Morgan et al., 2013). We refer to the measured horizontal shift of the psychometric curve as the ”empirical bias” (denoted by B in Fig. 3A), and we divide it into two components: (1) perceptual bias and (2) decision bias (see $P_{L},\;P_{R}$, and D in Fig. 3B). We refer to all biases that are not related to how the animal *perceives* the stimulus as ”decision biases,” which includes any biases in decision-making and motor planning/execution. Since the empirical bias reflects a combination of perceptual and decision biases, it is impossible to separate them using a simple 2AFC task (Fig. 3A).

However, if we have at least two experimental conditions, we can disentangle perceptual and decision biases (two unknowns) if we know how the biases in the two conditions relate to each other (two constraints) (e.g., see Linares et al., 2019). For instance, when multiple stimulus conditions are interleaved trial-by-trial, we can reasonably assume that the decision bias is the same across these conditions. Consider an experiment with two conditions in which the context-induced perceptual biases are expected to have equal magnitudes, but opposite signs. Then, subtracting the empirical biases measured in these two conditions will give an estimate of the perceptual bias that is not dependent on the common decision bias across the two conditions. We can also estimate perceptual biases with different magnitudes if we introduce a third, neutral condition in which context induces no perceptual bias. Consequently, for this neutral condition, we can assume that the empirical bias reflects only the decision bias. In this design, there are three conditions: one in which context induces a leftward perceptual bias, a second in which context induces a rightward perceptual bias, and a third neutral condition with no perceptual bias. The two perceptual biases can then be computed by subtracting the empirical bias measured in the neutral condition from the empirical biases measured in the other two conditions (Fig. 3B). This method can be generally used in any experiment involving a 2-AFC task with contextual modulations that produce different perceptual biases across two or more stimulus conditions.

Returning to our motion discrimination task example (Fig. 2), we can consider a situation in which there is leftward self-motion, no self-motion, or rightward self-motion, with all 3 conditions interleaved in a block of trials. In this case, it is reasonable to assume that the decision bias of the animal will be similar across the interleaved conditions. However, based on previous studies (Fajen & Matthis, 2013; Peltier et al., 2020; Rushton & Warren, 2005; Warren & Rushton, 2008, 2009a, 2009b), we expect rightward and leftward perceptual biases in the leftward and rightward self-motion conditions, respectively. In this case, we can estimate the perceptual biases caused by self-motion by subtracting the empirical bias measured in the no self-motion (i.e., neutral) condition from the empirical biases measured in the leftward and rightward self-motion conditions (Fig. 3B).

Building on this conceptual approach, we have developed a Bayesian model to estimate perceptual biases online during 2-AFC tasks (Fig. 3C, and see Methods 6.1). We model the decision-making process in the 2-AFC task as follows. First, as commonly done (Prins, 2023; Schütt et al., 2016), we assume that the percentage of making one of the two choices (e.g., a rightward response) follows a binomial distribution with parameter θ denoting the probability of choosing the first response alternative, and parameter n representing the number of trials. Second, similar to most previous methods (Peltier et al., 2020; Schütt et al., 2016), we used a cumulative Gaussian distribution as the functional form of the psychometric curve, reflecting the relationship between θ and the stimulus value, ω, (e.g., object motion direction). The two important parameters of the psychometric curve are (1) the sensitivity, S, which controls the slope of the psychometric curve (i.e., how rapidly θ changes as a function of ω), and (2) the empirical bias, B, which controls the horizontal shift of the psychometric curve (i.e., where the proportion of the two response alternatives are equal).

**Figure 4:**
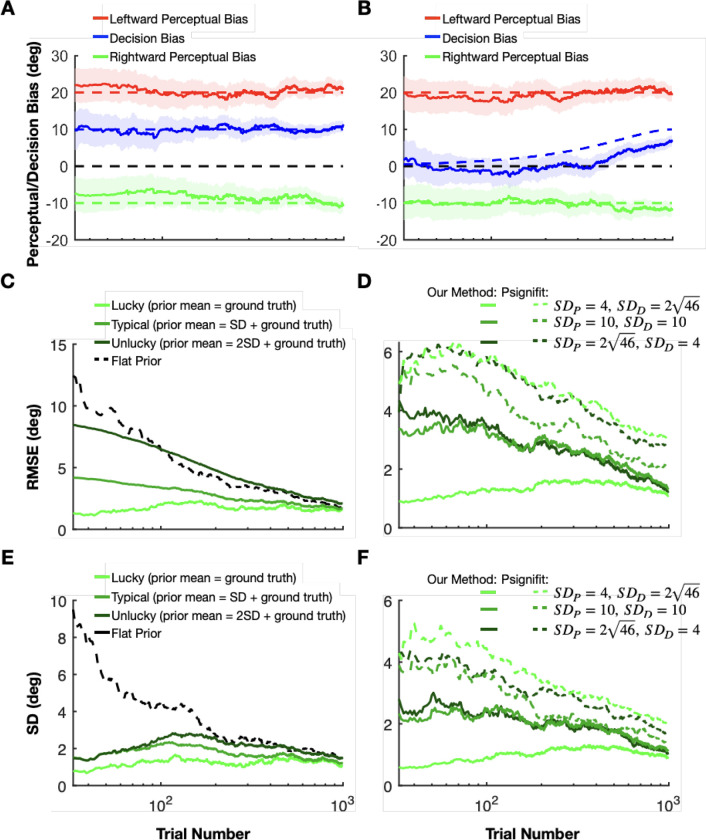
Validating our method with ground truth simulations. **A:** The mean (solid lines) and the SD (shaded areas) of the inferred perceptual (red in the rightward and green in the leftward self-motion conditions) and decision (blue, neutral, no self-motion condition) biases for an example synthetic data set. Ground truth perceptual bias was +20 in the leftward self-motion condition (dashed red) and −10 in the rightward self-motion condition (dashed green). The decision bias common to all conditions was +10, as seen in the neutral, no self-motion condition (dashed blue). **B:** The same as A, but showing an example synthetic dataset with a slowly changing decision bias. **C:** Average root mean square error (RMSE, y-axis), across 100 simulations, in estimating perceptual bias in the rightward self-motion condition, plotted as a function of trial number. Results are shown for three different prior mean values: 0, 1, and 2 standard deviations (SDs) away from the ground truth perceptual bias (from light to dark green, respectively). The black curve demonstrates results for a maximum likelihood estimator which corresponds to a Bayesian estimator with a uniform prior. **D:** Average root mean square error (RMSE, y-axis), over 100 simulations, in estimating perceptual bias in the rightward self-motion condition for three different values of prior widths for perceptual and decision biases (solid curves, light green to dark green, respectively). See text for details. Dashed curves show analogous results obtained using the conventional Bayesian Psignifit library. **E:** The same simulation as in **C**, but the SD of the perceptual bias averaged over 100 simulations is plotted. **F:** The same simulation in **D**, but the SD of the perceptual bias over 100 simulations is plotted.

Crucially, we further assume that the empirical bias, B, reflects the sum of the decision (D) and perceptual (P) biases. As mentioned earlier, we assume that all three conditions share the same decision bias; however, the perceptual bias is assumed to differ across self-motion conditions (Fig. 3B). Using a Bayesian framework, by selecting appropriate prior distributions for all root latent variables, including the perceptual biases, $P_{L}$ and $P_{R}$, in the leftward and rightward self-motion conditions, a common decision bias, D, and three separate sensitivities $S_{L},\;S_{N},$ and $S_{R}$, for the three self-motion conditions, we can achieve accurate estimates of the perceptual biases using a small number of trials. As more trials are added, the estimates of the biases become increasingly more accurate, as demonstrated below using synthetic data (Fig. 4).

Importantly, the use of a well-informed prior (based on past experiments) allows us to reward the animal based on an estimate of their perceptual bias from the beginning of the experiment, whereas inferring the bias based on a small number of trials would be wildly inaccurate without such a prior. At the beginning of each session, we gather data from the first 33 trials, each containing one data point from all unique stimuli. Using Bayesian updating, we combine the behavioral choices from these trials with our prior beliefs to compute an initial estimate of the posterior distribution over perceptual and decision biases. Then, we update our estimates of the posterior distributions over each bias based on the animal’s response in each subsequent trial. This way, we can estimate the perceptual bias of the animal trial-by-trial and flexibly update the reward boundary based on the estimated perceptual bias (see Methods 6.1 for more details and a formal description of the model).

### 3.3 Validation of the Algorithm Using Synthetic Data

To assess the validity of our method for estimating perceptual biases online in 2-AFC paradigms, we generated synthetic datasets simulating training sessions in the motion discrimination task with leftward self-motion, no self-motion, and rightward self-motion conditions. In line with the assumptions of our Bayesian model (see Methods 6.2 for more details), all biases were assumed to be stationary within a session (Fig. 4 A–E). We generated 100 synthetic sessions with different perceptual and decision biases, each with 1000 trials.

Consider a simulated dataset in which there are asymmetric perceptual biases for leftward and rightward self-motion, as well as a substantial decision bias (Fig. 4A). Our approach yielded estimates of both perceptual and decision biases that fluctuated within a reasonably narrow band around the ground truth values (Fig. 4A, dashed lines). As expected, the uncertainty bands around our estimates shrank continuously as the number of trials increased (Fig. 4A). Next, we quantified the accuracy of our method for three scenarios (Fig. 4C): in the first (“lucky”) one (light green), the ground truth perceptual bias coincides with the mean of the perceptual prior; in the second (typical) case (medium green), the ground truth is 1 standard deviations away from the prior mean, and in the third (“unlucky”) case (dark green), the ground truth is 2 SD away from the prior mean. For clarity, we present data only for the rightward self-motion condition (Fig. 4C); results for the leftward self-motion condition are analogous. As expected, the root mean square error (RMSE) grew with the mismatch between prior expectations and ground truth. Importantly, for the typical scenario, the RMSE was substantially lower than the error obtained by assuming a flat prior (maximum likelihood estimation) throughout the entire session (Fig. 4C, black). Additionally, for all of these cases, our method produces much less variable estimates of perceptual bias, as compared to using a flat prior (Fig. 4E), especially over the first 100 trials of a simulated session. The low variability of the estimates is crucial such that the reward boundary does not fluctuate wildly across trials.

A key difference between our method and state-of-the-art methods available through off-the-shelf libraries, such as Psignifit (Schütt et al., 2016), lies in our model’s ability to decompose the empirical bias into distinct decision and perceptual biases. This separation allows us to apply separate prior distributions to each type of bias, reflecting finer-grained knowledge from previous sessions or other subjects, for example. In contrast, employing a Bayesian method from an off-the-shelf library confines one to assigning priors solely to the empirical biases. Therefore, we assessed under what circumstances and to what extent our method outperforms the conventional Bayesian approach, as implemented using the Psignifit library (Schütt et al., 2016).

We ran our algorithm with three sets of prior distributions over the decision and perceptual biases (Fig. 4D, F). The mean values of these priors were consistently aligned with the actual ground truth. However, we varied the SD of the perceptual and decision biases across three scenarios. Initially, we set a broad SD for perceptual biases ($SD_{P}=\sqrt{46}\;degrees$) and a narrower one for the decision bias ($SD_{D}=4\;degrees$) (dark green in Fig. 4D, F). The second scenario involved equal SDs for both types of biases (10 degrees) (medium green in Fig. 4D, F). In the final case, the roles were reversed, with a narrow SD for perceptual biases ($SD_{P}=4\;degrees$) and a broader one for decision bias ($SD_{D}=2\sqrt{46}\;degrees$) (light green in Fig. 4D, F). Crucially, despite these variations in the width of the prior distributions, the empirical biases for both rightward and leftward conditions remained the same, centered around the ground truth values with an SD of $10\sqrt{2}$ degrees. Therefore, the conventional Bayesian methods would yield very similar estimates of empirical biases and their uncertainties across all scenarios both for leftward and rightward conditions. There will still be direct information about the decision bias from the neutral condition. Since the SD of the decision bias is different across the three scenarios it will affect the estimation of the perceptual bias. Nevertheless, we expect that our method will outperform conventional Bayesian methods when there is an informative prior on the perceptual biases. Indeed, we found that both the RMS errors (Fig. 4D) and the standard deviations (Fig. 4F) of the perceptual bias estimates were substantially lower for our method than for Psignifit, especially for the first 200 trials. The errors for our method were especially low in the case where the priors over the perceptual biases are narrow relative to the decision bias (light green curves). Consequently, our method has a considerable advantage over conventional Bayesian methods when the experimenter has a well-informed prior belief about the perceptual biases but does not know the decision-related bias of the animal before the training session.

We also tested how robust our method is when one of the assumptions of our Bayesian model is violated. Specifically, we tested scenarios in which only the perceptual biases were stationary over time, while the decision bias changed slowly within a session (Fig. 4 B). Interestingly, our method was robust against the slowly changing decision bias. The perceptual biases were estimated as accurately in the changing decision bias dataset as in the stationary decision bias dataset, and only the decision bias was systematically underestimated (Fig. 4B).

### 3.4 Application to Monkey Behavioral Data

**Figure 5:**
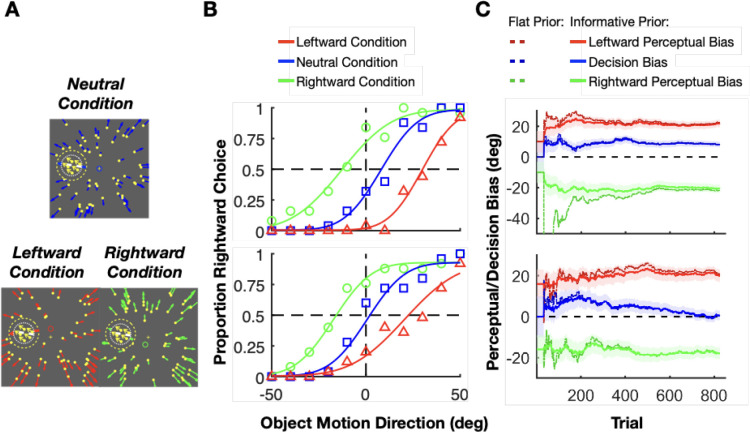
Applying our method to train monkeys in a motion discrimination experiment. **A:** Three experimental conditions with slightly different optic flow fields that simulated different forward self-motions. Yellow dots with colored arrows represent the background optic flow vectors. The dashed inner circle shows the location of the target patch, which contains moving dots (yellow dots with yellow arrows). The dashed outer circle represents a mask region within which no background dots appear. White dashed lines represent the implicit task reference around which the monkey had to discriminate the motion direction of the target patch. The small blue, red, and green rings represent the focus of expansion defining each of the three optic flow fields. Neutral condition: heading direction is forward (blue ring). Leftward condition: The heading direction is slightly upward (red ring), such that the optic flow vector at the target location is leftward of the reference. Rightward condition: The heading direction is slightly below and to the left of the center (green ring), such that the flow vector at the target location is rightward of the task reference. **B:** Psychometric functions from two example sessions (top and bottom), color-coded as in panel A. Smooth curves show fitted psychometric functions. **C:** The mean (solid lines) and the uncertainty (68% CI, shaded areas) of the inferred posteriors over perceptual (red, leftward condition; green, rightward condition) and decision (blue, neutral condition) biases across trials for the same two example sessions, using informative priors. Dashed lines: estimation using flat priors. We truncated the upper panel because the fat prior estimation for rightward perceptual bias is excessively negative. When using flat priors, estimates of the perceptual and decision biases show much larger fluctuations over the first few hundred trials (dashed lines). Actual reward was delivered based on the means inferred using informative priors.

We applied our method to reward monkeys during training in an experiment investigating motion perception with self-motion simulated by optic flow. The task of the monkey was to decide whether a patch of dots, referred to as the target, was moving rightward or leftward with respect to an implicit reference (Fig. 5A). We generated optic flow to simulate self-motion by displaying a full-field random-dot motion background. The discrimination boundary for our discrimination task (white dashed line in Fig. 5A) was aligned with the optic flow vector at the target’s location during simulated straight-forward translation (Neutral condition, Fig. 5A). As a result of this alignment, the categorical choices of an observer will be the same whether their percept is influenced by the optic flow or not. To estimate perceptual biases separately from decision-related biases using our method, the experiment involved three interleaved conditions (as suggested in Section 3.2). In the Neutral condition, optic flow simulated straight-forward translation, such that we expected their discrimination judgments to be unbiased (Fig. 5A, top). In the other two conditions, optic flow simulated slightly different heading directions (red and green circles, Fig. 5A, bottom), such that the optic flow vector at the location of the target would be slightly leftward (red) or rightward (green) of the discrimination boundary. Unlike the stimulus conditions that elicit optic flow parsing (Fig. 1B), here the optic flow produces an attractive perceptual bias rather than a repulsive bias, presumably because the optic flow is more closely aligned with the target motion. For our purposes here, we simply use these task conditions to illustrate the application of our method.

We trained monkeys to perform the motion discrimination task while using our method to estimate perceptual biases and deliver rewards. Monkeys showed a leftward perceptual bias in the Leftward condition and a rightward perceptual bias in the Rightward condition (i.e., attractive biases). Psychometric curves for two example sessions are shown in Fig. 5B. We used priors over the perceptual biases computed from data obtained in previous sessions (discussed further below). For the decision bias, we always used a prior centered at zero with a standard deviation estimated from previous sessions. In the first example session (Fig. 5C, top), the monkey’s perceptual biases turned out to be substantially greater than the prior mean (compare starting and ending values on the y-axis). Nevertheless, our Bayesian method quickly converged to a stable estimate of the perceptual biases, which enabled a stable reward schedule for the monkey with modest trial-to-trial variability. In the second example (Fig. 5C, bottom), we can observe a situation in which the decision bias of the monkey appears to change within the session. As shown in ground-truth simulations, our method appears robust against such changes in the decision bias (see Fig. 4E–H).

### 3.5 Integrating Hyperpriors to Combine Multiple Sessions with Varying Experimental Variables.

In the previous sections, we showed that our method can provide an accurate online estimation of perceptual and decision biases within a session. However, the performance of the algorithm is directly related to the strength of prior beliefs about the perceptual and decision biases. Had we used uninformative, uniform priors for estimating the biases, we would have observed substantial fluctuations in bias estimates over the first few hundred trials (dashed lines in Fig. 5C). Rewarding animals based on such wildly varying estimates of perceptual bias may confuse the animals and impair the progression of training. Therefore, it is important to understand how prior beliefs influence bias estimation in our method.

**Figure 6:**
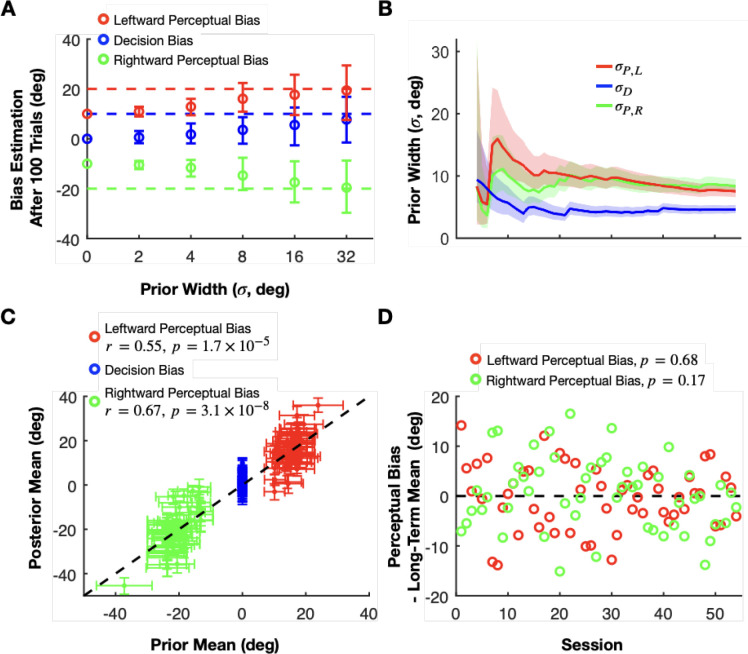
Importance of priors and hyperpriors in our method. **A:** Demonstrating how prior width influences the mean and variance of estimated bias values. The perceptual (red and green) and the decision (blue) biases were estimated after 100 trials in synthetic sessions with fixed ground truth biases (dashed colored lines) using priors with different widths (x-axis) but fixed means (colored circles at 0 width). The circles and error bars represent the means and the SDs across 20 simulations for each prior width, respectively. **B:** Dynamic narrowing of the estimated prior widths for the perceptual (green and red) and decision biases (blue) as the multi-session linear model integrates datasets from a progressively greater number of sessions (in chronological order). Solid lines and shaded areas represent the median and SD, respectively, of the prior widths for the perceptual and decision biases. **C:** The relationship between the prior means and the posterior means of the perceptual (green and red) and decision biases (blue). The prior means are estimated from the linear model using data from all sessions whereas the posterior means are inferred after integrating the monkey’s choices in each session. **D:** The differences between estimated priors and posteriors of perceptual biases across training sessions. Data are shown separately for leftward (red) and rightward (green) self-motion conditions. The data suggest that perceptual biases remain consistent throughout training with our reward method

To illustrate the consequences of being overconfident or underconfident, we generated another set of synthetic data with fixed perceptual and decision biases but with priors that have varying widths and fixed means centered on values that are 10 degrees away from the ground truth values. Specifically, the ground truth biases are $P_{L}=20,\;P_{R}=-20$, and D=10 in this simulation, whereas the corresponding prior means are 10, −10, and 0, respectively (Fig. 6A). The results of this simulation show the classic phenomena of bias-variance trade-off in statistics: narrower (overconfident) mismatched priors lead to low-variability estimates that are biased away from the true perceptual bias values, while wider (underconfident) mismatched priors result in less biased but highly variable estimates (Fig. 6A). Note that the error bars in Fig. 6A represent variability in the estimated mean biases across multiple simulated training sessions (with each simulated session having a different stochastic sequence of simulated choices), not uncertainty around the bias as in our other figures. Therefore, using well-chosen priors that are based on posterior beliefs after observing data from previous sessions is important for achieving a good balance between variability and accuracy.

Fortunately, our Bayesian method provides an optimal framework to combine data across sessions. Moreover, if some experimental variables that influence perceptual biases change between sessions (but remain constant within a session), we can extend our model by incorporating a hyperprior for these task variables. Taking the previously described motion direction discrimination task as an example, we observed that perceptual biases depend roughly linearly on heading direction (the focus of expansion of the optic flow), and the eccentricity of the target location (Fig. S2). Therefore, we modeled the priors over the perceptual biases for a session as Gaussian distributions centered around a weighted linear combination of heading direction and eccentricity. The weights in the linear combination can then be inferred with a hierarchical Bayesian model using data from multiple previous sessions with different heading directions and eccentricity values (see Methods 6.4, and Fig. S1 for more information). Thus, we use the information obtained from all previous training sessions, even though values of heading direction or eccentricity varies across sessions.

This approach allows us to select priors with optimal widths inferred from previous sessions for the online estimation in each subsequent session. As more sessions are completed, the uncertainty in the priors decreases and eventually converges to values that presumably reflect both the limited explanatory power of our simple linear model and any intrinsic variability in the animal’s perceptual biases from day to day that is not under experimental control (Fig. 6B). This provides us with a calibrated measure of uncertainty that is neither under- nor over-confident.

After applying a hierarchical multi-session model to all training sessions, we analyzed how well the inferred linear model can be used to estimate the biases in each session. We used the linear weights from the extended model, estimated using data from all sessions, to compute the mean of the perceptual bias prior for each session, representing our estimated perceptual bias before the session begins. Across sessions, these prior estimates (shown on the x-axis in Fig. 6C) are positively correlated with the posterior estimates of perceptual biases (Pearson correlation: leftward perceptual bias: *r* = 0.55, *p* = 1.7 × 10^−5^; Rightward perceptual bias: *r* = 0.67, *p* = 3.1 × 10^−8^), which are updated after integrating all of the choices made by the monkey within each session (y-axis in Fig. 6C). What this means is that our linear model for combining data across sessions accounts for about a third of the variance in perceptual bias. The remaining unexplained variance is due to variables other than eccentricity and heading direction that are either knowable to the experimenter and could therefore be included in an improved model, or unknowable like internal brain states appearing like random variability. Importantly, our Bayesian model accounts for these by the width of the prior representing our lack of knowledge about the true perceptual and decision biases before the session. Regarding the decision bias, we hypothesized that it is not influenced by heading direction or eccentricity. Thus, we always used a prior centered around zero for the decision bias with a variance reflecting its session-to-session variability. We found that inferred posteriors over the decision biases were centered around zero with an SD of about 5 degrees (blue error bars in Fig. 6C).

The central motivation for our work is to reward the animal in such a way as to not alter its intrinsic perceptual biases. Thus, a critical test of our approach is to examine whether an animal’s perceptual bias is stable across time. We can do so by comparing the perceptual bias predicted by our stationary, time-independent, linear model (prior mean) with the perceptual bias obtained from the actual responses (posterior mean) (Fig. 6C). A diminishing perceptual bias over time would be indicated by a negative trend in the differences between posterior means and prior means across sessions. Conversely, a stable perceptual bias would manifest as fluctuations around a difference of zero. A linear regression analysis on these differences relative to session numbers revealed no significant trends in either leftward (*p* = 0.17) or rightward perceptual biases (*p* = 0.68), demonstrating that the perceptual biases remained stable through the course of training with our reward method.

## 4 Discussion

We propose an adaptive method to reward animals for reporting their subjective (biased) percepts in 2-AFC tasks. Our methodology allows neuroscientists to study neural mechanisms of subjective percepts, across long periods of time, without the reward scheme inducing biases into those subjective percepts, or into the animal’s reporting of their percepts, due to the animal’s desire to maximize reward. Our method infers the perceptual bias of the animal separately from other decision-related biases after each trial and rewards the animal based on the estimated perceptual bias. We used a hierarchical Bayesian framework to optimally integrate data from previous sessions, thereby improving the accuracy of the bias estimation and reward allocation across multiple training sessions. Using extensive ground-truth simulations, we demonstrated the accuracy and precision of our approach. We applied our method to train monkeys in a motion perception task with unknown subjective percept, demonstrating its effectiveness in estimating perceptual biases. Crucially, we demonstrate that the monkey’s perceptual biases are stable across over 50 training sessions using our reward method, in stark contrast to results from the same animal in a previous study (Peltier et al., 2020). These findings pave the way for future studies of the neural basis of subjective percepts in animals that require thousands of trials, even when those percepts are not known *a priori* and need to be inferred themselves.

### 4.1 Rewarding animals when stimulus context biases perception

Several reward strategies have been used in previous work to mitigate the issue of reward allocation in trials for which a perceptual bias is expected (e.g., an illusion). The most straight-forward approach involves rewarding animals veridically based on the stimulus (Parrish et al., 2015; Tudusciuc & Nieder, 2010). Studies employing this method typically train the animal extensively in conditions without perceptual biases. Then, they run limited test trials in the probe (i.e., illusion) conditions for which perceptual biases are expected, under the assumption that rewards will not alter the animal’s reports. However, if many probe trials need to be presented, the lack of rewards for specific stimuli incentivizes animals to change their decision strategy to maximize the reward rate (see Fig. 2). Another strategy used in some prior studies was to give no reward in probe conditions for which a perceptual bias was expected (Agrillo et al., 2015; Agrillo et al., 2014; Clara et al., 2006). However, given the need for large numbers of trials in neuroscience studies, this strategy could demotivate animals, particularly in tasks (e.g., in Fig. 5) for which many conditions are expected to induce perceptual biases. A third strategy is to always reward animals in conditions with perceptual biases (Bayne & Davis, 1983; Fujita, 1997). This strategy is also problematic for experiments requiring extensive trial counts because animals may learn that they can give any random response in the conditions for which the experimenter wants to measure perceptual biases. Several other studies employed a strategy intermediate between the previously mentioned strategies: rewarding animals randomly in 80% (Barbet & Fagot, 2002, 2007) or 50% (Huang et al., 2002) of trials with expected perceptual biases. Again, this strategy could lead to demotivation and random responses when the animal experiences large numbers of trials in conditions that evoke perceptual biases.

Researchers could also reward animals based on a prediction about the expected magnitude and sign of the perceptual biases in their experiment. For example, we could reward animals for reporting motion in world coordinates in the direction discrimination task depicted in Fig. 1. However, this strategy might simply ”train in” a particular decision-related bias based on reward feedback, rather than revealing the animal’s intrinsic perceptual bias. To avoid introducing artificial decision biases while still motivating the animals, some studies have also tried rewarding animals randomly (Huang et al., 2002; Peltier et al., 2020) or consistently (Fang et al., 2019) around their *predicted* perceptual bias. A problem with this approach is that the extent of an animal’s perceptual bias typically cannot be predicted *a priori*. Indeed, this method resulted in gradually decreasing perceptual biases over many training sessions in a previous study involving motion discrimination (Peltier et al., 2020).

In contrast, we propose that the best strategy is to infer the perceptual biases of animals from their responses online and to allocate rewards by aligning the reward boundary with the estimated perceptual bias. Since the algorithm converges toward the ground truth bias rather quickly when the prior estimate is inaccurate, a mismatch between the intrinsic perceptual bias and the reward boundary does not persist for long periods of time. Our results show that the monkey’s perceptual bias remained stable over 50+ training sessions (Fig. 6D), indicating the effectiveness of our strategy. In contrast, data from the same animal in a previous study (Peltier et al., 2020), which attempted to reward animals randomly based on a prediction of the perceptual bias, showed perceptual biases that declined substantially over a similar time period.

### 4.2 Separating perceptual and decision biases

One of the most challenging aspects of rewarding animals based on their subjective perception is that one needs to distinguish the animal’s perceptual biases from other decision-related biases. Since it is impossible to dissociate perceptual from decision-related biases in simple 2-AFC tasks (Drugowitsch et al., 2016; Garcia-Pérez & Alcalá-Quintana, 2013; Jogan & Stocker, 2014; Morgan et al., 2013; Shivkumar et al., 2022), previous studies also interleaved different conditions (Jazayeri & Movshon, 2007; Linares et al., 2019; Sánchez-Fuenzalida et al., 2023; Zamboni et al., 2016), presented a variable number of stimuli before the subject responded (Drugowitsch et al., 2016), or used multimodal stimuli (Shivkumar et al., 2022) to decompose the sources of the empirically measured response bias. Other previous studies intermixed discrimination and estimation tasks trial by trial to measure perceptual biases directly using the estimation task (Jazayeri & Movshon, 2007; Zamboni et al., 2016). A couple of studies used serial dependencies (Cicchini et al., 2017; Fritsche et al., 2017) or the tilt surround illusion (Cicchini et al., 2021) to separate perceptual from decision-related processes using only estimation tasks. In a recent study, researchers combined all of the aforementioned approaches, and interleaved estimation and discrimination tasks about line lengths while also manipulating the base rate of the stimuli and the visual context with the Muller-Lyer illusion (Sánchez-Fuenzalida et al., 2023). This allowed the authors to quantify the extent to which the illusion affected perceptual and decision-related processes. Our approach is similar in spirit to methods that interleave conditions with different contexts to elicit different perceptual biases. However, in contrast to the previously mentioned studies, our objective was not to reveal a perceptual process in a specific experiment but to use the inferred perceptual biases to devise a method for rewarding animals to report their subjective percepts in 2-AFC tasks in order to study the perceptual biases’ neural basis.

### 4.3 Bayesian methods for estimating psychometric functions

A challenging aspect of rewarding animals based on their subjective perception is that the estimates of biases need to be fairly accurate and stable from the beginning of a training session when only a small number of trials have been completed. Therefore, we developed a hierarchical Bayesian method that can combine data from previous sessions even if some of the experimental variables, such as eccentricity or heading direction, vary across sessions (see Fig. 6). This allows us to optimally combine our prior knowledge about the perceptual biases with the information coming in from the animal’s response after each trial.

Several previous studies have implemented Bayesian inference for estimating psychometric functions in 2-AFC tasks (Frund et al., 2011; Kattner et al., 2017; Prins, 2023; Roy et al., 2021; Schütt et al., 2016). However, those previous methods were not designed to infer perceptual biases separately from decision biases but rather to estimate the empirical bias. The primary focus for a subset of these studies was estimating a dynamically changing psychometric function online (Bak et al., 2016; Frund et al., 2011; Kattner et al., 2017; Roy et al., 2021), while for other studies the emphasis was on efficient estimation of a more stable psychometric function given all of the responses in the experiment (Prins, 2023; Schütt et al., 2016). In contrast, we aimed to estimate the perceptual biases of animals separately from other decision-related biases, in an online fashion, during the experiment, to reward the animal in real time.

Furthermore, we sought to combine data across sessions for which we needed a method that could apply separate prior distributions over the perceptual and decision biases. In contrast, previous methods (e.g., Prins, 2023; Schütt et al., 2016) only allow applying priors to empirical biases. Our approach, which uses separate prior distributions for perceptual and decision biases, proved to be significantly more accurate in estimating perceptual biases than Psignifit (Schütt et al., 2016), a popular off-the-shelf method that only allows for priors over empirical biases. This difference was especially large when one’s prior over the perceptual biases was stronger than one’s prior over the decision bias (see Fig. 4D, F). Please note that we are not claiming that our algorithm is better at estimating psychometric functions than previous methods. We only claim that using separate priors for perceptual and decision biases results in a more accurate estimation of the biases.

Finally, a previous study proposed an optimal, adaptive training algorithm for animal experiments to speed up the training procedure (Bak et al., 2016). Although their method also involves estimating psychometric functions in 2-AFC tasks online, in contrast to our method, they aimed to maximize the learning rate of the animals which involved selecting a mixture of easy and difficult stimuli that decrease all biases and history dependencies of the animals (Bak et al., 2016). Importantly, their approach assumes that the correct answer is known for each stimulus, and does not address the issue of how one would estimate perceptual biases that are unknown *a priori*.

### 4.4 Limitations

The main assumption of our method is that the perceptual and decision biases of the animal are stable within a session. Furthermore, we assume that perceptual biases remain stable across sessions, which could be reasonably assumed from analogous human perception studies (Bekrater-Bodmann et al., 2012; Cretenoud et al., 2021), such that integrating data across sessions to establish well-informed priors is the optimal strategy. However, in the case of experiments where the perceptual biases of the animal are expected to change during training, our method could be adapted to account for such non-stationarities within and across sessions. For example, perceptual biases might change in studies investigating perceptual learning, which is the refinement of perceptual processes after extensive training on perceptual tasks (Chen et al., 2016; Hua et al., 2010; Schoups et al., 2001; Yan et al., 2014). In such a case, there are several possible ways to modify our method. The simplest is to use a sliding window for determining the history of data that is used for estimating the perceptual and decision biases (X.-G. Gao et al., 2013). Alternatively, one can apply a Gaussian process prior over the biases (e.g., Bak et al., 2016; Roy et al., 2021), or define a function *apriori* capturing the expected temporal evolution of the biases (e.g., Kattner et al., 2017). However, these adjustments come with trade-offs. More complicated models with more parameters typically need more data for accurate estimations. Thus a Gaussian process, with its less restrictive prior, or a sliding window approach, which relies on smaller data sets, will likely introduce greater noise into the inference process, potentially diminishing the effectiveness of the method.

Regarding decision biases, a more realistic assumption is that the decision strategies of the animals can change within a training session resulting in a change in decision bias (Ashwood et al., 2022). On the one hand, our method is robust to a slowly changing decision bias (see Fig. 4B). On the other hand, our method would need adjustments for experiments in which the decision strategies of the animals are expected to change rapidly. For example, at the beginning of training, before animals reach stable performance levels, they tend to explore different decision strategies and exhibit strong decision biases (Gold et al., 2008). However, in most studies, animals would likely be trained to reasonably stable performance on a task condition for which perceptual biases are not expected, and our method might then be employed once other task contexts are introduced that induce perceptual biases.

## 5 Conclusion

Studying the neural basis of perceptual biases poses a substantial challenge in neuroscience. Rewarding animals to report their subjective percepts, which may be different from the presented stimulus, is difficult because the experimenter has no direct access to the animal’s subjective percepts. We propose that the best strategy is to infer animals’ subjective percepts online from their responses and allocate rewards based on the estimated perceptual bias. We implemented a hierarchical Bayesian framework that can provide a real-time, trial-by-trial estimation of perceptual biases and can also combine data across multiple sessions with variable task conditions. Data from one monkey trained to perform a motion discrimination task demonstrate stable perceptual biases over many sessions when employing our reward strategy.

## 6 Methods

### 6.1 Hierarchical Bayesian Model

To model the trial-by-trial decision-making process in the motion direction discrimination task, we assign a probability of choosing the rightward choice, θ, for a stimulus direction, $\omega_{m}$, in a contextual condition, k, using a cumulative Gaussian distribution, Φ:

$$\theta_{m,k}=\Phi \left(\omega_{m}|B_{k},S_{k}\right)$$

The parameters $B_{k}$ and $S_{k}$ correspond to the Gaussian distribution’s mean and standard deviation and describe the observer’s empirical bias and sensitivity, respectively. Importantly, this probability $\left(\theta_{m,k}\right)$ is not solely determined by the stimulus on the retina, $\omega_{m}$, but is also influenced by contextual information in condition k, leading to different empirical bias, $B_{k}$, and sensitivity, $S_{k}$, parameters in each different contextual condition.

The empirical bias, $B_{k}$, comprises both perceptual, P, and decision, D, biases in general. In our example with three contextual conditions, we have three empirical biases, $B_{L},\;B_{N}$ and $B_{R}$, along with three sensitivities, $S_{L},\;S_{N}$, and $S_{R}$, for the leftward, neutral, and rightward self-motion conditions. The three contextual conditions are interleaved trial-by-trial. We assume that the empirical biases in the three contextual conditions are the following combinations of perceptual and decision biases:

$$B_{L}=P_{L}+D$$

$$B_{N}=D$$

$$B_{R}=P_{R}+D$$

where $P_{L},\;P_{R}$, and D represent the leftward perceptual bias, rightward perceptual bias, and decision bias, respectively. We assume that the perceptual ($P_{L}$ and $P_{R}$) and the decision (D) biases are stationary within a session. We further assume that the decision bias (D) doesn’t change across the interleaved contextual conditions.

We used Gaussian prior distributions for the perceptual and decision biases:

$$P_{L}∼N\left(P_{0,L},\tau_{P_{L}}\right)$$

$$P_{R}∼N\left(P_{0,R},\tau_{P_{R}}\right)$$

$$D∼N\left(D_{0},\tau_{D}\right)$$

where $P_{0,L},\;P_{0,R}$, and $D_{0}$ represent the means of the prior distributions, reflecting our best estimates based on prior knowledge obtained before the data was collected (as discussed further below). The standard deviations $\tau_{P_{L}},\;\tau_{P_{R}}$, and $\tau_{D}$ represent the uncertainty associated with these estimates.

We used a gamma prior distribution for the sensitivities:

$$S_{L}∼\Gamma (\alpha ,\beta )$$

$$S_{R}∼\Gamma (\alpha ,\beta )$$

$$S_{N}∼\Gamma (\alpha ,\beta )$$

where we used the same parameters, α and β, for the sensitivities in all contextual conditions since it is unlikely that the sensitivity of the animal changes across the interleaved contextual conditions we used in our experiment. Note that if, in other experiments, α and β most likely change across the conditions, our method allows us to use different α and β parameters for each experimental condition also.

We used lapse rates, accounting for the tendency to make incorrect choices due to guessing:

$$\theta_{m,k}=\lambda_{1,k}+\left(1-\lambda_{2,k}-\lambda_{1,k}\right)\Phi \left(\omega_{m}|B_{k},S_{k}\right)$$

where $\lambda_{1,k}$ and $\lambda_{2,k}$ represent lapse rates for leftward and rightward choices, respectively. We assume that all lapse rates λ follow a Beta distribution with the same parameters, γ, and ϵ:

$$\lambda_{1,k}∼Beta(\gamma ,\epsilon )$$

$$\lambda_{2,k}∼Beta(\gamma ,\epsilon )$$

Finally, we assume the animal’s choices follow a Bernoulli distribution:

$$C_{t}∼Bernoulli\left(\theta_{m,k}\right)$$

### 6.2 Model Validation

We validated our method using ground-truth simulations. We generated synthetic data for a motion direction discrimination experiment with 3 self-motion conditions. We generated the target patch’s (dashed inner circles in Fig. 5A) motion directions ranging from −50 to 50 degrees (deg.) with steps of 10 deg., resulting in a total of 11 target motion directions. Similar to the real experiment (see Methods 6.3), the stimuli in the simulations were generated with block randomization, ensuring that each stimulus direction was shown once before repeating the same set of stimuli. With three contextual conditions (leftward, neutral, and rightward self-motion), this meant that each block contained a randomized order of 11 (motion directions) × 3 (contextual conditions) trials. In each synthetic session, we generated 30 blocks of trials resulting in 990 trials in total (30 blocks × 11 motion directions × 3 contextual conditions = 990 trials). The monkey’s choices were generated using the model described in the previous section (Methods 6.1) with different ground truth combinations of perceptual and decision biases. We assumed zero lapse rates in the simulations.

First, in line with the assumptions of our Bayesian model (see Methods 6.1), we generated data with a stationary decision bias equal to 10 deg. (see Fig. 4 A,B,C & E). Second, we generated data with a decision bias that changed slowly within a session following a sinusoidal function, D=10sin(πn/2000), where n represents the trial number (see Fig. 4 B). The ground truth perceptual bias was always 20 deg. for the leftward contextual condition (see dashed red lines in Fig. 4A, B). The perceptual bias in the rightward contextual condition was always −10 deg., which is half the magnitude of the leftward perceptual bias with an opposite sign (see dashed green lines in Fig. 4A, B). The ground truth value for the sensitivity was consistently set to 15 deg. across all conditions and simulations, which approximated the slopes of psychometric functions observed in the monkey behavior during our experiment.

We used Gibbs sampling using the STAN Matlab toolbox (Carpenter et al., 2017; Stan Development Team, 2022a, 2022b) to compute the posteriors over the perceptual and decision biases ($P_{L},\;P_{R}$, and D), the sensitivities ($S_{L},\;S_{N}$, and $S_{R}$), and the lapse rates ($\lambda_{1,k}$ and $\lambda_{2,k}$). We generated 5000 samples, of which we discarded the first 2500 (burn-in). In each session, the inference via Gibbs sampling started after the first 33 trials (i.e., after one repetition of all stimulus directions in each condition). Then, the posteriors over all variables in the model were updated after every subsequent trial. We applied the same Gibbs sampling algorithm to estimate the posteriors in the monkey training sessions.

To compute the posteriors, we used the same priors over the sensitivities, S∼Γ(8,0.5), and the lapse rates, λ∼Beta(1,10), in all three contextual conditions in each simulation. However, we tested our method with different sets of priors over perceptual and decision biases. We independently generated 100 synthetic sessions for each combination of priors over perceptual and decision biases we tested.

First, we tested how different offsets between the prior mean and the ground truth values of the perceptual bias influence the estimation result (Fig. 4C, E). We used prior means zero, one, and two standard deviations from the ground truth values for the perceptual biases while fixing the prior over the decision bias to be centered at zero. The standard deviations for perceptual and decision biases were fixed at 5 and 10 deg., respectively. We also included a maximum likelihood estimation version of the model, corresponding to Bayesian estimation with a uniform prior. Since the quantity we are estimating is an angular variable, we chose to use a uniform prior with the range −180 to 180 deg.: $P_{L}∼U(-180,\;180)$, D∼U(−180,180), $P_{R}∼U(-180,\;180)$.

Second, we compared our method to conventional Bayesian methods using the Psignifit library (Schütt et al., 2016), while applying different ratios of prior widths between perceptual and decision biases (Fig. 4D, F). Thus, we estimated the biases using our method described in the previous section and using the Matlab library of Psignifit (Schütt et al., 2016). The main difference between Psignifit and our method is that one cannot apply priors separately to perceptual and decision biases when using Psignifit – only over the aggregated empirical biases. To make the results of the Psignifit implementation comparable with our method, we used the same priors for the empirical biases for both methods. The prior over the empirical bias in the neutral condition in the Psignifit implementation was chosen to be equal to the prior over the decision bias in our method, $B_{N}∼D∼N(0,\;10)$. However, the empirical biases in the leftward and the rightward conditions are sums of perceptual and decision biases. Thus, the priors over the empirical biases in those conditions can be computed by convolving the priors over the decision and perceptual biases. This leads to the following distributions of the empirical biases: $B_{L}∼N(P_{L}+D,\sqrt{\tau_{P_{L}}^{2}+\tau_{D}^{2}}),\;B_{R}∼N(P_{R}+D,\sqrt{\tau_{P_{R}}^{2}+\tau_{P_{R}}^{2}})$. In the simulations comparing our method to Psignifit, we used the ground truth values for the prior means and the following prior widths for the perceptual and decision biases: (1) $\tau_{P_{L}}=\tau_{P_{R}}=2\sqrt{46},\;\tau_{D}=4$; (2) $\tau_{P_{L}}=\tau_{P_{R}}=10,\;\tau_{P_{D}}=10$; (3) $\tau_{P_{L}}=\tau_{P_{R}}=4,\;\tau_{P_{D}}=2\sqrt{46}$.

### 6.3 Motion Discrimination Task with Optic Flow

We trained one monkey to perform a motion discrimination task in which a patch of random dots moved in a specific direction with 100% coherence (arrows in the dashed inner circles in Fig. 5A). After a 2-second stimulus period, the monkey had to indicate their decision by making a saccadic eye movement within 1 second. The direction reference (white dashed line in Fig. 5A) was implicitly indicated by the locations of the two saccade targets (not shown in Fig. 5A). The monkey would receive a juice reward for making a saccade to the correct target determined by our online rewarding method. Across behavioral sessions, the location of the target patch varied extensively, and the direction reference covaried accordingly.

We simulated contextual information (mimicking self-motion) using optic flow to investigate whether the monkey’s perception of object motion is attracted toward the optic flow vector that would exist at the location of the target patch. In the neutral condition, optic flow simulated forward self-motion, and we implicitly assigned the direction reference for the discrimination to the local optic flow direction in this condition (white dashed line is aligned with the blue local motion vector in Fig. 5A, top). We did not anticipate any bias in the psychometric curve in this neutral condition. In the other two conditions, the heading directions (colored circles in Fig. 5A, bottom) were shifted along an implicit circle around the screen’s center point with a radius equal to the eccentricity of the target patch. The optic flow vectors had the same length in all three conditions, but their angles differed across the contextual conditions (Fig. 5A).

In the two example sessions shown in Fig. 5B, C, we used priors computed from previous sessions based on the extended hierarchical model (see the next section). In the top subplots in Fig. 5B, C, we used the following priors: $P_{L}∼N(-10,\;7)$, $P_{R}∼N(10,\;7)$, and D∼N(0,5). In the bottom subplots in Fig. 5B, C, we used the following priors: $P_{L}∼N(-16,\;7)$, $P_{R}∼N(16,\;7)$, and D∼N(0,10). The psychometric curves in Fig. 5B show the empirical biases estimated from all trials in the session.

### 6.4 Extended Hierarchical Bayesian Model

We extended our model in section 6.1 with a simple linear model with additional latent variables capturing how the perceptual bias changes due to varying heading directions and the eccentricities of the stimuli across the sessions (Fig. S1). We simply assumed that the heading directions and eccentricities have a linear relationship with the perceptual biases:

$$P_{R}=Xw_{R}$$

$$P_{L}=Xw_{L}$$

where X denotes the task variables that change across sessions, which in our case is a matrix with heading direction and eccentricity values in columns, and w denotes the weights. Therefore, the prior over the perceptual biases can be written as follows:

$$P_{R}∼N\left(w_{R}X,\sigma_{R}\right)$$

$$P_{L}∼N\left(w_{L}X,\sigma_{L}\right)$$

We used uninformative hyperpriors over the variables, $w_{R},\;w_{L},\;\sigma_{R}$, and $\sigma_{L}$, which were estimated across sessions since we usually have limited prior information for these variables. In Fig. S2, we show the measured relationships between the perceptual bias and heading directions and the eccentricities of the stimuli.

In Fig. 6C, D, we inferred $w_{R},\;w_{L},\;\sigma_{R}$, and $\sigma_{L}$, using all 53 sessions’ of monkey behavioral data. In each session, we use these inferred posteriors over $w_{R},\;w_{L},\;\sigma_{R}$, and $\sigma_{L}$ (using all sessions) together with the session-specific values of X to construct the session-specific priors over the perceptual and decision biases. Then, we estimate the posteriors (y-axis, Fig. 6C) over the perceptual and decision biases using these session-specific priors (x-axis, Fig. 6C).

## Supplementary Material

Supplement 1

## Data Availability

The source data of the motion discrimination experiment is publicly available in the reward perception GitHub repository, https://github.com/GaborLengyel/reward_perception.
