# Supplementary material for: How to reward animals based on their subjective percepts: A Bayesian approach to online estimation of perceptual biases
Source: bioRxiv. 2025 Mar 7:2024.07.25.605047. Originally published 2024 Jul 25. Preprint. [Version 2] doi: 10.1101/2024.07.25.605047 (PMC11291170; doi:10.1101/2024.07.25.605047)
Supplement: Supplement 1 [file NIHPP2024.07.25.605047v2-supplement-1.pdf]

bioRxiv preprint doi: https://doi.org/10.1101/2024.07.25.605047; this version posted March 7, 2025. The copyright holder for this preprint (which was not certified by peer review) is the author/funder, who has granted bioRxiv a license to display the preprint in perpetuity. It is made available under aCC-BY 4.0 International license.

9 Supporting information

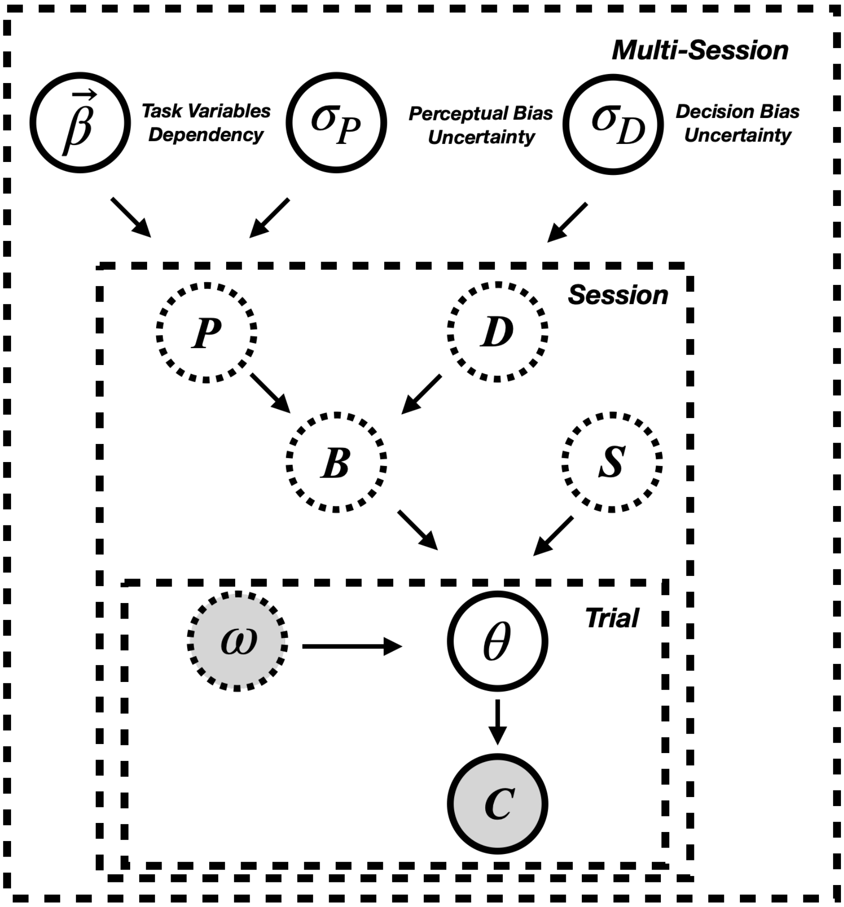

Figure. S1: **Extended generative model with hyperpriors.** The two inner plates represent the same generative model as in Fig. 4C. This model is extended by incorporating latent variables shared across sessions. The vector $\vec{\beta}$ encodes the weights that determine how perceptual biases vary with heading direction and stimulus eccentricity across sessions. σ_P and σ_D represent the standard deviations of the perceptual and decision bias random variables, respectively. See Methods 6.9 for further details.

bioRxiv preprint doi: https://doi.org/10.1101/2024.07.25.605047; this version posted March 7, 2025. The copyright holder for this preprint (which was not certified by peer review) is the author/funder, who has granted bioRxiv a license to display the preprint in perpetuity. It is made available under aCC-BY 4.0 International license.

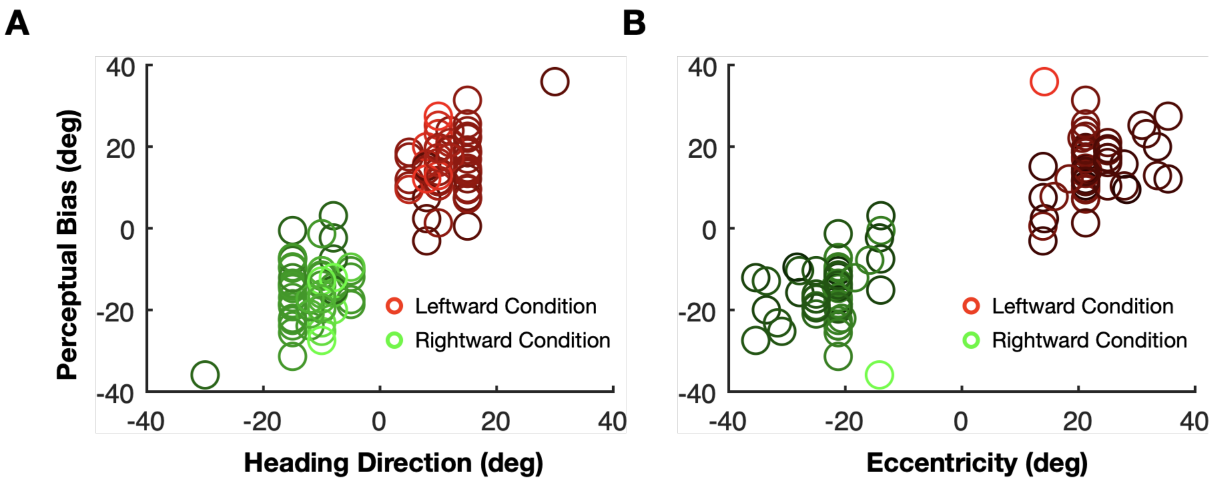

Figure. S2: **The relationship between perceptual biases, heading direction, and object eccentricity A:** Relationship between perceptual bias and the heading direction simulated by optic flow in the monkey task. With a larger heading direction, the monkey showed a larger perceptual bias in leftward and rightward conditions. Symbol colors, from light to dark, represent eccentricity, from large to small. **B:** Relationship between perceptual bias and object eccentricity. With a larger eccentricity, the monkey showed a larger perceptual bias in leftward and rightward conditions. Symbol colors from light to dark represent heading directions from large to small.

bioRxiv preprint doi: https://doi.org/10.1101/2024.07.25.605047; this version posted March 7, 2025. The copyright holder for this preprint (which was not certified by peer review) is the author/funder, who has granted bioRxiv a license to display the preprint in perpetuity. It is made available under aCC-BY 4.0 International license.

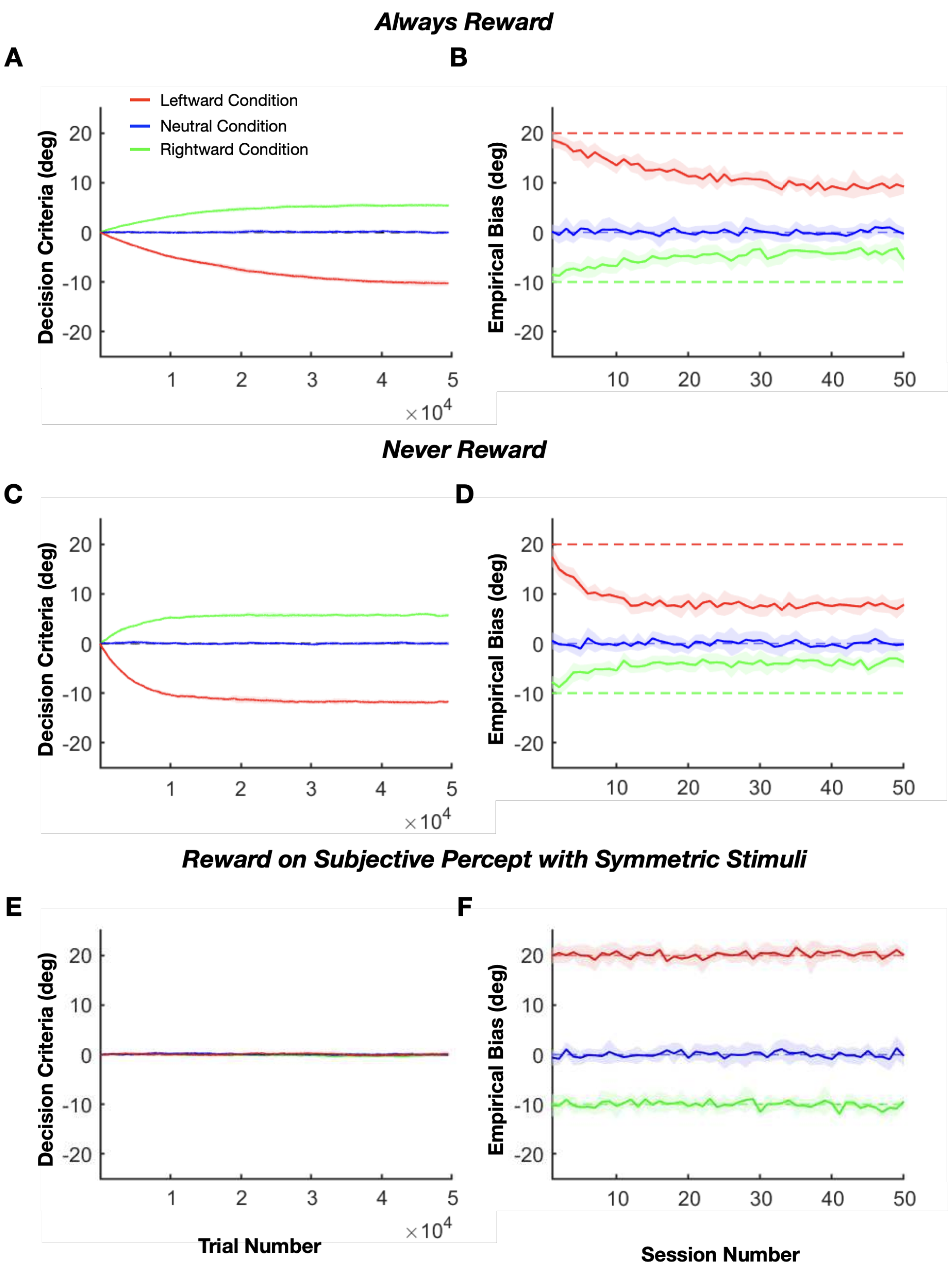

1277

Figure. S3: textbfAdditional RL agent simulations. **A, B:** Same as Fig. 3A, B, but with an 'always reward animals in ambiguous trials' strategy. **C, D:** Same as Fig. 3A, B, but simulating a 'never reward animals in ambiguous trials' strategy. Note that results for both the 'always' and 'never' reward strategies are quite similar to those of the random reward strategy shown in Fig. 3C, D. **E, F:** Results from an RL agent simulation in which reward is based on the ground truth perceptual biases, as in Fig. 3E,F. The only difference is that, in this simulation, the range of object directions was symmetrical around the true perceptual bias for each of the contextual conditions specified by optic flow. In this case, decision criteria remain the same across contexts, indicating that the small separation observed in Fig. 3E results from stimulus range effects.

bioRxiv preprint doi: https://doi.org/10.1101/2024.07.25.605047; this version posted March 7, 2025. The copyright holder for this preprint (which was not certified by peer review) is the author/funder, who has granted bioRxiv a license to display the preprint in perpetuity. It is made available under aCC-BY 4.0 International license.

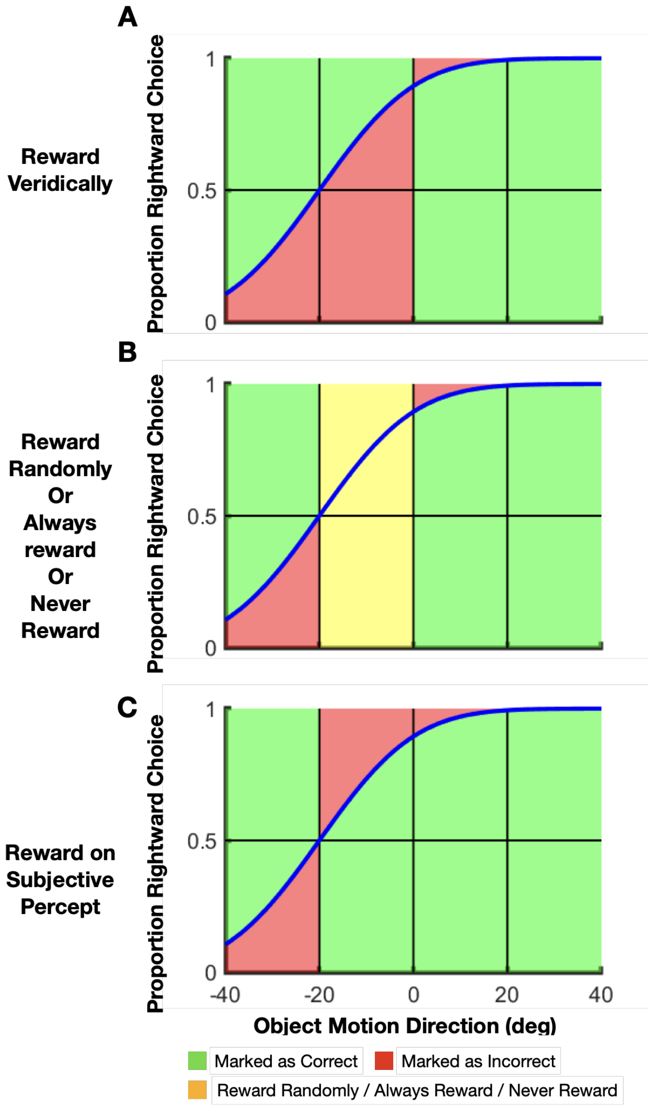

Figure. S4: textbfDiagram of choice outcomes for different reward paradigms. The blue curve represents an example psychometric curve, with perceptual bias $P = -20$ deg and slope $S = 16$. Shading indicates the proportions of trials that are scored as correct (green) or incorrect (red), as well as trials that are rewarded differently in different methods (yellow). **A:** Rewards are based on veridical stimulus value, such that the reward boundary is at zero object direction despite the perceptual bias. **B:** Yellow shading indicates a range of stimulus values for which it is assumed that the "correct" answer cannot be known (i.e., there is an illusion). Within this range, rewards are delivered randomly, always, or never. **C:** Rewards are based on the animal's subjective percept, such that the reward boundary is aligned with the true perceptual bias of $P = -20$ deg. Note that the animal will receive more total reward (less red area) as compared to the veridical reward strategy.

bioRxiv preprint doi: https://doi.org/10.1101/2024.07.25.605047; this version posted March 7, 2025. The copyright holder for this preprint (which was not certified by peer review) is the author/funder, who has granted bioRxiv a license to display the preprint in perpetuity. It is made available under aCC-BY 4.0 International license.

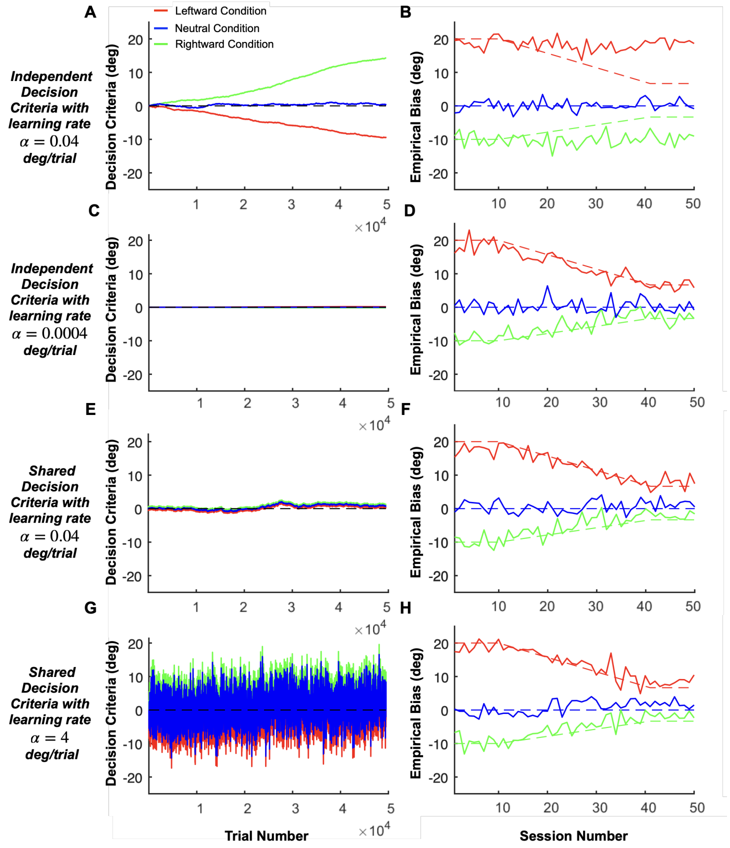

Figure. S5: textbfRL agent simulations for slowly-changing perceptual biases. Each panel shows 50 simulated sessions, with each session comprising 990 trials. **A, B:** The RL agent adjusts independent decision criteria for each optic flow condition, with a moderately fast learning rate of 0.4 deg/trial. **A:** Solid curves depict the learned decision criteria across the three contexts: leftward (red), rightward (green), and neutral (blue) self-motion. The dashed black line (barely visible behind the solid blue line) represents zero decision criterion. **B:** Solid curves show the estimated empirical biases in the three self-motion conditions. Red and green dashed lines show how the ground truth perceptual biases change over time, starting at +20 and -10 deg for leftward and rightward self-motion, respectively, and decreasing linearly to $20/3$ and $-10/3$ deg before stabilizing. In this case, the RL agent could adjust its decision criteria faster than our method could track the changing perceptual biases, causing a mismatch between the measured (solid) and true (dashed) biases. **C, D:** Same as **A, B**, but with the RL agent having a much slower learning rate. In this case, our method accurately tracks the changing perceptual biases. **E, F:** Same as **A, B**, but with an RL agent restricted to having a single decision criterion that is shared across contextual conditions. Since our method estimates perceptual biases independently of any decision biases that are shared across conditions, it performs effectively in this scenario. **G, H**: Same as **E, F**, but with a much faster learning rate of 4 deg/trial. If the decision criterion is shared across contexts, our method works well even with very fast learning rates.

bioRxiv preprint doi: https://doi.org/10.1101/2024.07.25.605047; this version posted March 7, 2025. The copyright holder for this preprint (which was not certified by peer review) is the author/funder, who has granted bioRxiv a license to display the preprint in perpetuity. It is made available under aCC-BY 4.0 International license.

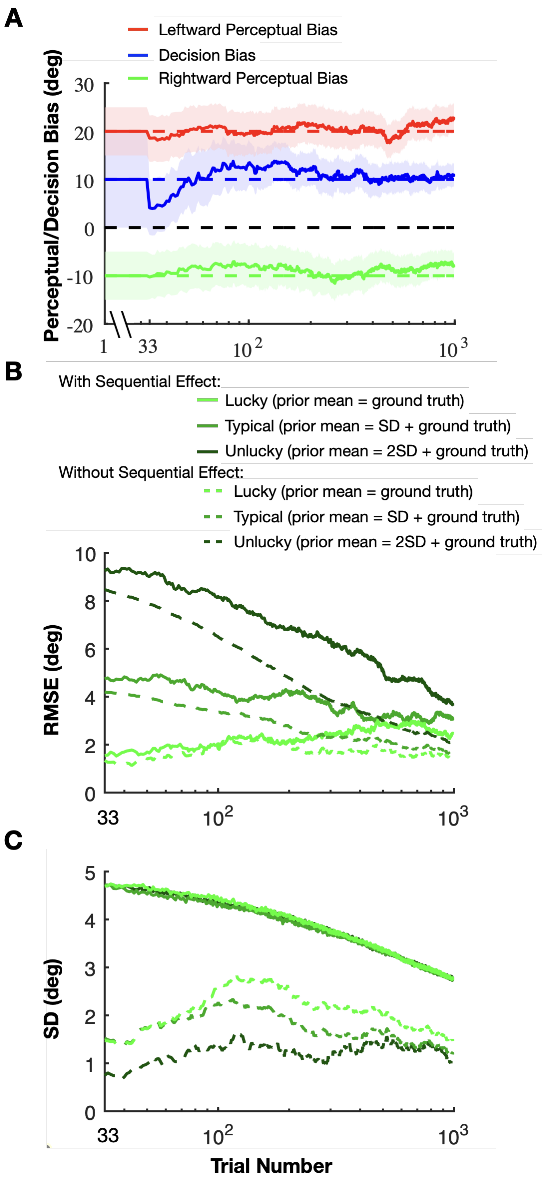

Figure. S6: **Assessing our method's robustness to the presence of sequential choice effects A:** A ground-truth simulation very similar to Fig. 6A & B, but with built-in sequential choice effects. On each simulated trial, the agent is given a 25 % probability of using a win-stay-lose-shift strategy, repeating the previous choice when rewarded and switching to the alternative choice when not rewarded, regardless of the stimulus values. Note that our method still tracks the ground-truth perceptual biases in the presence of the choice history effect. **B:** Solid curves: Average root mean square error (RMSE, y-axis), across 20 simulations, in estimating perceptual bias in the rightward self-motion condition, plotted as a function of trial number. Results are shown for three different prior mean values: 0, 1, and 2 standard deviations (SDs) away from the ground truth perceptual bias (from light to dark green, respectively). Dashed curves: replotted from the simulation of Fig. 5C, which didn't include any sequential choice effects. When a sequential effect is present, the RMSE shows a slight increase but remains relatively small, indicating that the model's performance is still adequate. **C:** Analogous result to panel B, but showing the average standard deviation (SD) of perceptual bias estimates as a function of trial number. Again, performance is similar, but SDs are somewhat larger in the presence of choice history effects.
