# Supplementary material for: How to reward animals based on their subjective percepts: A Bayesian approach to online estimation of perceptual biases
Source: bioRxiv. 2024 Jul 25:2024.07.25.605047. Preprint. [Version 1] doi: 10.1101/2024.07.25.605047 (PMC11291170; doi:10.1101/2024.07.25.605047)
Supplement: Supplementary file 1 [file NIHPP2024.07.25.605047v1-supplement-1.pdf]

bioRxiv preprint doi: https://doi.org/10.1101/2024.07.25.605047; this version posted July 25, 2024. The copyright holder for this preprint (which was not certified by peer review) is the author/funder, who has granted bioRxiv a license to display the preprint in perpetuity. It is made available under aCC-BY 4.0 International license.

857 # 9 Supplementary figures

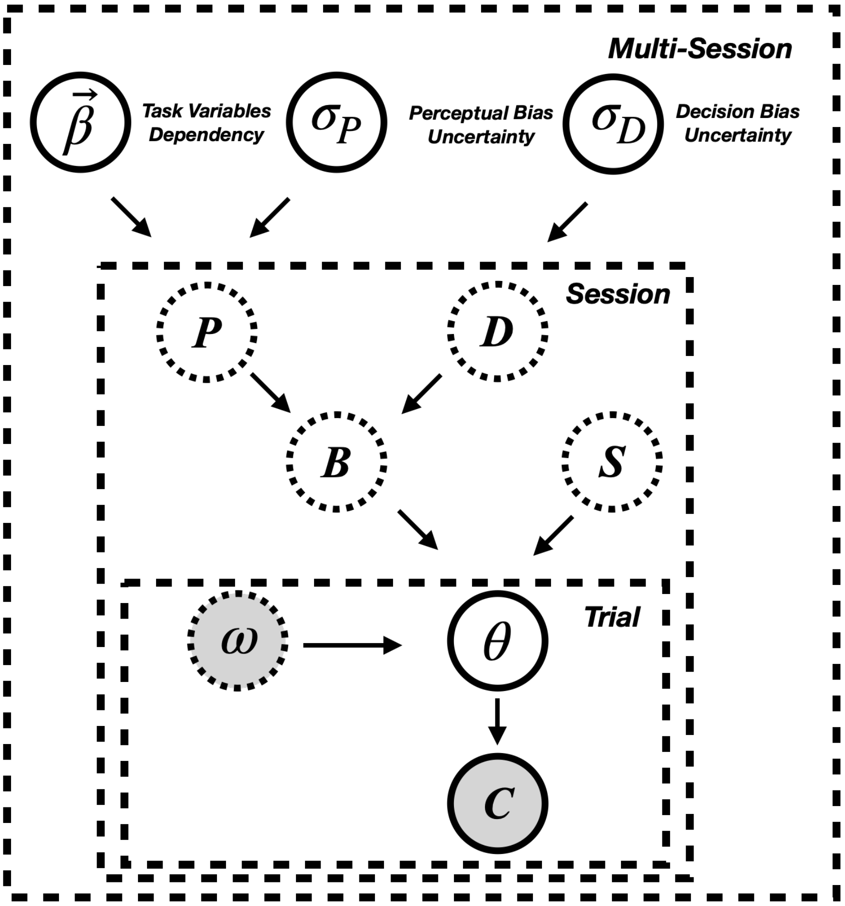

Figure. S1: **Extended generative model with hyperpriors.**

bioRxiv preprint doi: https://doi.org/10.1101/2024.07.25.605047; this version posted July 25, 2024. The copyright holder for this preprint (which was not certified by peer review) is the author/funder, who has granted bioRxiv a license to display the preprint in perpetuity. It is made available under aCC-BY 4.0 International license.

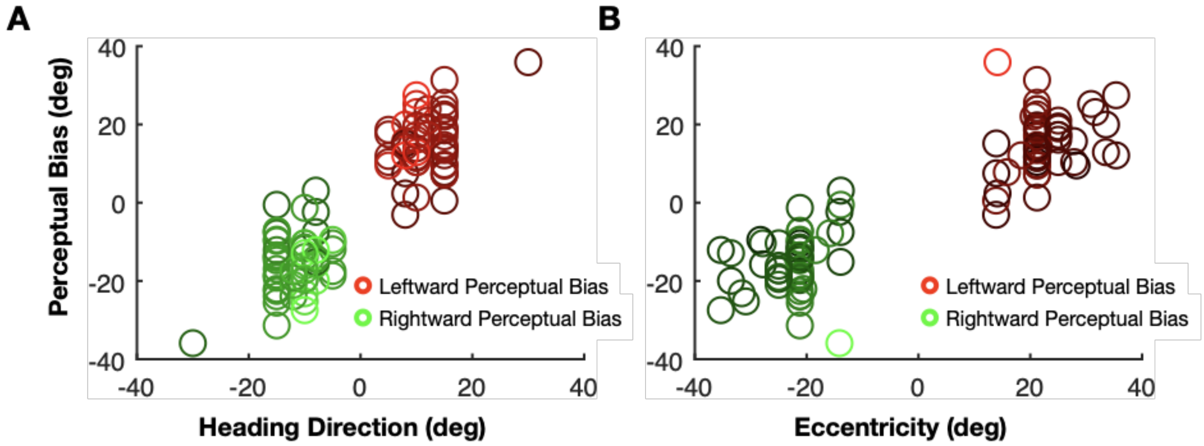

Figure. S2: **The relationship between perceptual biases heading direction and the eccentricity A:** Relationship between perceptual bias and heading direction. With a larger heading direction, the monkey showed a larger perceptual bias in leftward and rightward conditions. Circles' color from light to dark represents the eccentricity from large to small. **B:** Relationship between perceptual bias and object eccentricity. With a larger eccentricity, the monkey showed a larger perceptual bias in leftward and rightward conditions. Circles' color from light to dark represents the heading direction from large to small.
